# Supplementary figures and images for: Robust repression of tRNA gene transcription during stress requires protein arginine methylation
Source: Life Sci Alliance. 2019 Jun 3;2(3):e201800261. doi: 10.26508/lsa.201800261 (PMC6549136; doi:10.26508/lsa.201800261)

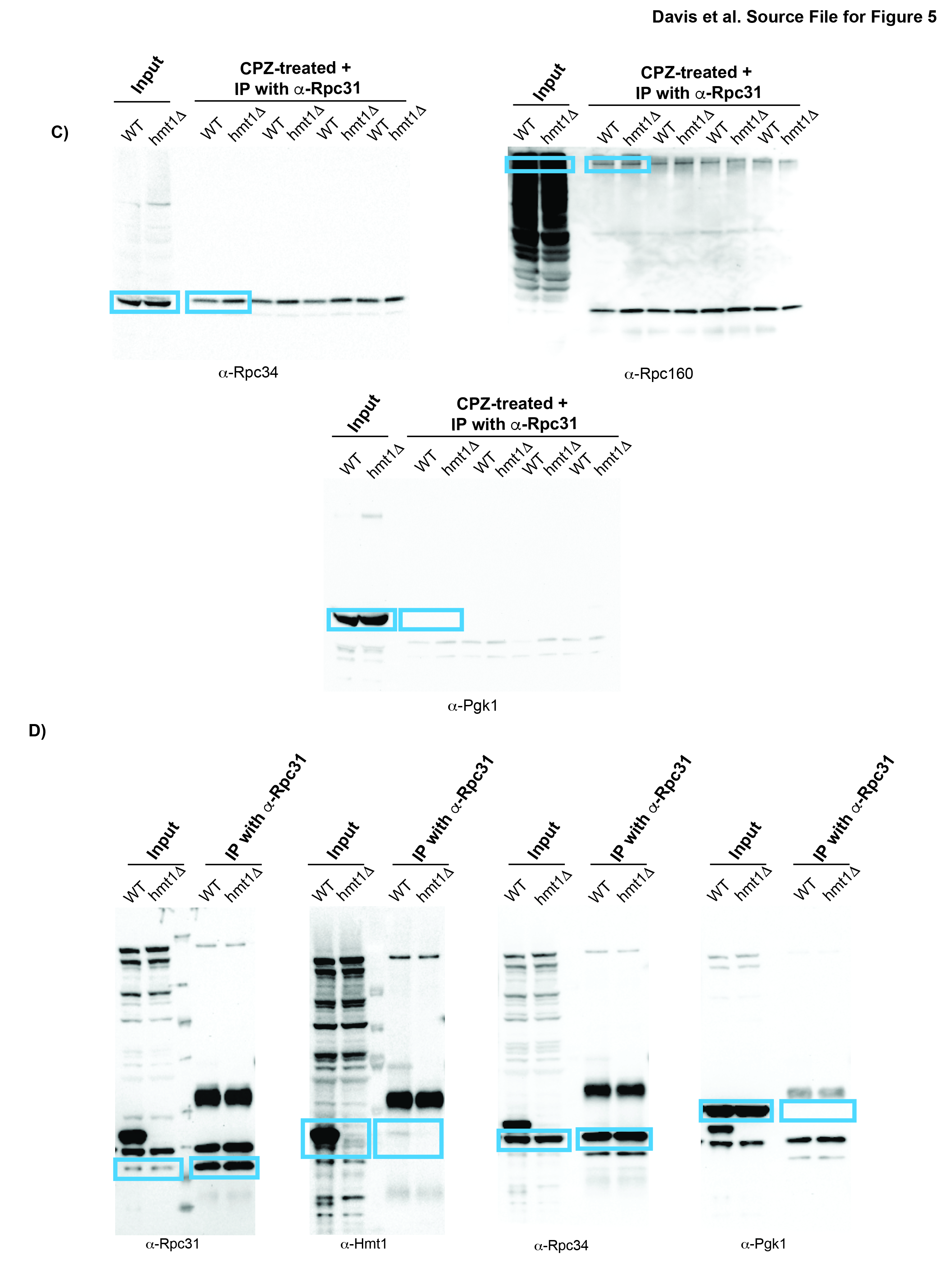

Supplement: Supplementary file 2 [file LSA-2018-00261_Sdata5C,D.tif]
